# Apoptosis in Colorectal Cancer

**Published:** 2014-06-25

**Authors:** M Stoian, N State, V Stoica, G Radulian

**Affiliations:** * "Carol Davila" University of Medicine and Pharmacy, Bucharest, Internal Medicine Department, "Dr. Ion Cantacuzino" Clinical Hospital, Bucharest; ** “Carol Davila" University of Medicine and Pharmacy, Bucharest, Institute of Diabetes, Nutrition and Metabolic Diseases “N. C. Paulescu", Bucharest

**Keywords:** apoptosis, colorectal cancer, oncogene bcl-2, colorectal mutagenesis, tumoral necrosis factor alpha

## Abstract

Abstract

Apoptosis is an inborn process that has been preserved during evolution; it allows the cells to systematically inactivate, destroy and dispose of their own components thus leading to their death. This programme can be activated by both intra and extracellular mechanisms. The intracellular components involve a genetically defined development programme while the extracellular aspects regard endogenous proteins, cytokines and hormones as well as xenobiotics, radiations, oxidative stress and hypoxia. The ability of a cell to enter apoptosis as a response to a “death" signal depends on its proliferative status, the position in the cell cycle and also on the controlled expression of those genes that have the capacity of promoting and inhibiting cell death. The fine regulation of these parameters needs to be maintained in order to ensure the physiological environment required for the induction of apoptosis. Any malfunction in any of the steps of controlled cellular death can lead to dysfunctions and, as a consequence, to different pathological conditions. The importance of apoptosis lies in its active nature and in the potential of controlling biological systems.

## Introduction

Apoptosis or programmed cell death is essential for the development and for the well functioning of multicellular systems. In order to ensure functional and structural tissue homeostasis non-necessary and deteriorated cells have to be removed from an otherwise healthy cellular microclimate. Examples of apoptosis have been observed in almost all cellular types during their development and maturation. 

 Throughout embryonic life, a genetically controlled programme that involves cell proliferation and apoptosis supervises the genesis of cells and organs. 

 In adult tissues, physiological cell death appears in cyclic stimulated tissues or hormonally dependent tissues like the endometrium, the prostate or the mammary gland, but also in the steady-state turnover of many other tissues. Selective cell death is fundamental for the development, regulation and for the well functioning of the immune system including the elimination of auto-reactive thymocytes, the negative selection of T and B-lymphocytes and also for the cellular death induced by cytotoxic T-lymphocytes. Endogenous processes that have the capacity of detecting irreparable genetic deteriorations are involved in the process of limiting the dissemination of these abnormalities. The lymphocytes invading immunologically "privileged" tissues are rapidly eliminated, thus offering protection from a possible inflammatory response in these special [**1**]. Nowadays, the unified concept behind all these processes is that apoptosis is mediated by a common set of events and it uses similar biochemical pathways leading to a stereotypic disposition of the structural alterations. Apoptosis is an inborn process that has been preserved during evolution; it allows the cells to systematically inactivate, destroy and dispose of their own components thus leading to their death. This programme can be activated by both intra and extracellular mechanisms. The intracellular components involve a genetically defined development programme while the extracellular aspects regard endogenous proteins, cytokines and hormones, as well as xenobiotics, radiations, oxidative stress and hypoxia. The ability of a cell to enter apoptosis as a response to a “death" signal depends on its proliferative status, the position in the cell cycle and also on the controlled expression of those genes that have the capacity of promoting and inhibiting cell death. The fine regulation of these parameters needs to be maintained in order to ensure the physiological environment required for the induction of apoptosis. Any malfunction in any of the steps of controlled cellular death can lead to dysfunctions and, as a consequence, to different pathological conditions. 

 The importance of apoptosis lies in its active nature and in the potential of controlling biological systems.

 Cellular death has been considered a chaotic process. Nevertheless, just as a single cell can balance between anabolic and catabolic states, the entire organism needs to balance the proliferation and cell death states in order to ensure homeostasis. Overall, an organism needs to dispose of aged, deteriorated or abnormal cells that have the potential of malignant transformation. As such, the changes that may appear in the regulation of apoptosis could contribute to the pathogenesis of degenerative and neoplasic diseases. Although physiological cell death has been described for many decades, the problem has been put forward again in the seventies when Kerr, Wyllie and Currie have described in detail the infrastructural changes characteristic of dying cells and have also proposed the term apoptosis to define this process. They have showed that physiological cell death is not a random process but a process that possesses distinct morphological features. It usually affects individual cells and once initiated it evolves rapidly. 

 The ingestion of apoptotic cells by macrophages does not induce the release of proteolytic enzymes or active oxygen species by the latter cells. The fragmenting of the dying cells takes place without an extracellular leak of their contents and the removal of these cells does not induce inflammation. The absence of inflammation represents a crucial feature of apoptosis, thus allowing cell death without an alteration of the adjacent structures. 

**Genetics and apoptosis**

 Despite numerous research advances, it is still very difficult to identify all the molecules involved in the apoptosis process in mammalian organisms. Fortunately, the nematode Caenorhabditis elegans represents a particularly useful study model of the genetic regulation of apoptosis [**2**]. Throughout the embryonic and larval stage of its development, a number of 131 of a total of 1090 cells are being eliminated following a very constant and well characterized genetic programme. Thus, a large number of mutations have been identified and the responsible genes have been ordered to form a genetic map. 

 These genes are involved in the decision to enter apoptosis, in incorporating dying cells in mononuclear cells, in the execution of this process and in degrading the cellular debris. Two of these genes, ced-3 and ced-4 are necessary in order for all the forms of apoptosis to occur and it is thought that they encode the final effectors of this path. Another regulatory key gene, ced-9, is involved in suppressing apoptosis in those cells that are pre-programmed to live [**3**]. This gene encodes a protein that is homologous to the Bcl-2 gene from humans. Moreover, the expression of Bcl-2 can inhibit apoptosis in nematodes and can even partially substitute the loss of function of Ced-9, indicating that at least some parts of the apoptosis have been conserved during evolution. There are mutations appearing in six genes that are responsible for the uploading of apoptotic bodies into non-professional "neighbor" cells. Intracellular proteins like Ced-2, Ced-5 and Ced-10 use signaling pathways similar to those of other mammalian homologues like Crkll, DOCK 180 and Rac thus modulating cytoskeleton’s reorganization and extension in the incorporating cell. Ced-7, homologous to ABC-1 is activated in both the apoptotic cell and in the macrophages. Ced-1 is analogue to the scavenger receptors in the mammalian system; Ced-7 and Ced-1 are probably involved in promoting the uploading process through their interaction with the signaling adaptor protein Ced-6 [**4**]. 

 There are 14 different genes that suffer mutations affecting apoptosis at different levels. Only a small number of genes are affected by mutations influencing the decision to enter the cell death process. The next steps are similar to those seen in any somatic cell engaged in apoptosis. The activation of ced-3 and ced-4 promotes cell death while ced-9 is able to prevent this process. 

**Colorectal mutagenesis and the cellular apoptotic response **

 The malignant transformation of colorectal polypoid adenomas begins with mutations in the nuclei of the epithelial colorectal cells. Human diet contains numerous mutagenic agents including substances that are metabolized into mutagens. Most of these are chemical toxins synthesized by plants as an immediate response to any form of injury caused by bacteria, insects, fungi or other microorganisms. Because the presence of these components is so obvious, the colonic mucosa contains effective innate mechanisms of defense. 

 The metabolism of precarcinogenic substances is a complex process that involves the following events: intestinal absorption, liver metabolization, bile secretion and colonic oxidative processes. Some studies have used dimethylhydrazine (DMH) to show the malignant transformation of colorectal polyps. DMH is a procarcinogen absorbed and then oxidized and hydrolyzed in the liver generating a less stable compound called metilazoximetanol (MAM), which is then involved in the alteration of DNA methylation. The host cell responds by attempting to repair the damaged DNA and if this process does not succeed, the last option is to enter apoptosis. 

 Dwan, Meier and Blackman have shown genetic differences in the DMH induced colorectal cancer model. They have observed that not all the mice species develop bowel tumors in response to DMH and that there is a potential induction of leukemic neoplasia when using this substance. 

 After the administration of DMH, the colonic epithelium develops tumors even if this epithelium is transplanted on small bowel structures. This process does not occur when small bowel tissue is transplanted on the colonic mucosa. A single dose of DMH is enough to determine colorectal adenomas and carcinoma after only four months. By the 15th week of treatment with DMh the characteristic G-A mutations appear in 66% of the k-ras genes; the whole process takes about 24 weeks to finalize. When one of these mutations leads to the inactivation of a vital gene, the cell is then eliminated. Only one cell needs to be affected by mutations in codons 12, 13 and 61 of the regulatory sequence of k-ras because this cell will then have the capacity to multiply and to invade the surrounding tissue. This genetic model reinforces the sequential nature of genetic lesions and it also proves that the initial specific mutations in colorectal carcinogenesis are determined by chemical carcinogens. 

**The significance of apoptosis in cancer and in cancer therapy **

 The implications of apoptosis in pathology 

 The disruption of apoptosis leading to excessive cellular death constitutes the basis of the initiation and progress of many human diseases. It is thought that an exacerbation of apoptosis in neural cells could be involved in the progression and severity of many conditions such as Alzheimer’s and Parkinson’s disease, amyotrophic lateral sclerosis, spinal muscular atrophy and even stroke. Excessive apoptosis of circulating T cells is related to AIDS while the opposite of this process contributes to the development of many autoimmune and lymphoproliferative disease. 

 Neoplasia is nowadays considered the result of either the inhibition of apoptosis, by the loss of functional mutations in cell death activation genes, or the acquirement of such mutations in cell death suppressor genes. Viruses have developed mechanisms to manipulate apoptosis in order to multiply; these include inhibiting apoptosis in the infected cells and at the same time stimulating apoptosis of the defense mechanisms in the host. Taking into account these facts, the manipulation of apoptosis by genetic or pharmacological intervention will probably allow the development of new treatments for a multitude of diseases. 

 The circumstances involved in the development of apoptosis can be divided into two categories: apoptosis in normal tissues and in specific pathological states. At least part of the latter. The cells can respond to a potential lesion by apoptosis. If a drug induces apoptosis in a lymphocyte, this usually gives us more information about the cell and not about the drug itself so, this sort of experiments need to be interpreted cautiously. The therapeutic implications are that a drug that has the capacity to inhibit apoptosis can be used for the prevention of cell death while other forms of therapy will remove the infection. 

 On the contrary, in cancer states, the therapeutic value will consist in the ability to stimulate apoptosis in malignant cells. The way of obtaining this effect with adequate specificity is still a challenge. Researchers have to take a close look at the nature’s own killer cell that is the cytotoxic T lymphocyte. 

 Some other therapies are represented by antibodies that have the capacity to trigger apoptosis and also by immunotoxins that can “hide" apoptosis inducing ligands. 

 It took four million years in the evolution process to develop an outstanding system capable of eliminating unwanted cells; it is now up to researchers to discover the way to set this device on the “on" or “off" position on demand. 

 The characteristic intercellular liaisons in evolved life species would not have been possible without an effective mechanism of removing no longer necessary or malfunctioning cells. Defective regulation of programmed cell death could play a role in the etiology of cancer, AIDS, autoimmune and degenerative CNS conditions. Pharmacological manipulation of apoptosis offers new potential prophylactic and treatment opportunities. 

**Malignant transformation **

 Because a cell needs to suffer a series of alterations in order to achieve a malignant phenotype and because agents or treatments that eventually often induce these changes, alter the cells, any other element that will activate the survival of deteriorated cells has the capability to promote carcinogenesis. 

 In most instances, there are morphologic differences between normal and cancer cells. The nucleus is often enlarged, hypo or hyperchromic, with an irregular nuclear membrane, prominent single or multiple nucleoli. There is also an increase in the nucleus to cytoplasm ratio. All of these changes represent morphologic signs of malignancy. There are also exceptions to this rule: in some forms of cancer, the malignant cells are quite similar to normal cells. This makes the diagnosis of malignancy very difficult and some other architectural criteria need to be applied. 

 The majority of cancers are characterized by the fact that they contain large numbers of dividing cells. These mitoses are often abnormal. The mitotic index defined by the number of mitoses per microscopic unit is of a special value because it gives information on the prognosis of different tumors. In reality, the mitotic index in cancers is not as high as that observed in non-tumoral pathology. Still, in some forms of cancers, the diagnosis of malignancy relies on this concept [**5**]. 

**Apoptosis and cancer**

 The traditional interest in oncology has been cell proliferation. Nowadays, the main concern is the apoptosis process [**6**]. 

 The presence of apoptosis in tumors is not a recent observation. Over twenty years ago, it has been suggested that programmed cell death is responsible for many of the cell losses that appear in tumors. It is no longer news the fact that apoptosis is induced by irradiation, chemotherapy, hormonal manipulation or other therapeutic strategies. 

 Numerous research studies conducted in the past years on the molecular control of apoptosis have led to a better understanding of the oncogenic potential of this process. For example, the discovery of the possible regulation of apoptosis by certain proto-oncogenes and by the suppressor tumor gene p53 has opened the road for new research. 

 Malignant transformation is the result of an abnormal number of cells that have been modified at the DNA level. The tumor growth speed is determined by the ratio between cell death and cell division. Certain cancer cells divide slower than normal cells but malignant changes continue to expand because of prolonged lifespan of that certain cell. 

 There are many carcinogens that cause ruptures in the DNA material or interfere with the enzymes involved in the correct replication of DNA. The cell can answer to this aggression in many ways: it can delay the division of that specific cell until the lesion is repaired, it can enter apoptosis or it can evolve without any intervention in the cell cycle. 

 Apoptosis is an effective measure of preventing malignant transformation because it can eliminate potentially altered cells. Nevertheless, it can also promote cancer growth by either allowing the accumulation of dividing cells but also by blocking the removal of certain genetic variants that have a high malignant potential. It is not known yet what determines the cell to enter one of the three pathways underlined above: entering apoptosis after a lesion has been detected, repairing that lesion or continuing with the normal cell cycle. Paradoxically, some genes that have the potential to stimulate cell division like c-myc are involved in the “opening" of that specific path. RNA concentrations and c-myc level rise early in apoptosis and it has been shown that overexpression of these two components can induce apoptosis in fibroblasts [**7**]. 

 The oncogene bcl-2 could be regarded as “the general suppressor of cell death" among those genes that are directly involved in the regulation of apoptosis. Every hematopoietic cell, many epithelial and neural cells contain this protein mostly in the mitochondrial membrane, the nucleus and the endoplasmic reticulum. For example, follicular B cell lymphomas have a high concentration of the bcl-2 protein because of a specific translocation [t(14:18)] which places the bcl-2 gene under the strict control of the promoter of the gene that encodes immunoglobulin heavy chain. Epstein-Barr viral proteins are involved in raising the level of bcl-2 in Burkitt lymphoma cells [**8**]. 

Compared to normal B-lymphocytes, the cells that express bcl-2 are able to survive for longer periods in cultures that are not enriched with growth factors and these cells are resistant to both ionizing radiations and glucocorticoids. It has also been shown that high bcl-2 levels can protect from c-myc induced apoptosis [**9**]. Transgenic mice engineered to overexpress bcl-2 protein in B-lymphocytes often develop diffuse and large cell lymphomas. Half of these tumors contain a translocation of the c-myc gene [**10**]. 

 The protein derived from p53 suppressor gene is able to delay the progression of cell cycle before the initiation of DNA synthesis. Many human cancers contain deletions or mutations of this specific gene. There are also many viral proteins that can bind and inactivate the p53 gene. The malfunctioning of this key element promotes cancer growth by allowing DNA replication in altered cells before they have undergone the repair process. Apart from the fact that p53 inhibits cell division; it can also act as a direct “apoptogene". As such, the overexpression of the normal p53 protein in a myeloid leukemia cell line will rapidly induce apoptosis and cell death [**11**]. 

 Many cell types require for proliferation at least two external stimuli that are called the competence and the progression signals. The first can launch metabolic events that are common to both apoptosis and replication; the progression signal will turn the cell to the replication process, otherwise it will enter apoptosis. 

 Programmed cell death can be considered an alternative for eliminating those cells that fails to pass the “checkpoint" before DNA replication and it takes place in competent activated cells whenever a progression signal misses, is abnormal or when a cell is not able to repair its lesion in a certain timeframe. 

 Most of the antitumor drugs such as topoisomerase inhibitors, alkylating agents, antimetabolites and antagonist hormones induce apoptosis in susceptible cells. Based on these facts, it is easily understood that their effect is not always related only to the interaction with a biochemically specific target; influencing different metabolic events, which can induce apoptosis and can also interfere with the response to chemotherapy. It is known that in various human cancers, the concentrations of different calcium dependent endonucleases are not constant so that the degradation of DNA throughout apoptosis is not a stable process. The susceptibility for apoptosis in cancer cells is important in the therapy of particular malignancies that have a low rate of cell growth and are particularly unresponsive to chemotherapy. 

**Spontaneous apoptosis in tumors**

 Apoptosis can be found in virtually all untreated tumors. There have been few precise quantitative studies, but histologic evaluation points out the fact that the extent of this process in some human tumors resembles the one observed in tissues suffering from a rapid involution, thus underlining its kinetic significance. 

 Programmed cell death is often present in locations where it coexists with necrosis and where it is supposed that moderate ischemia is involved in its initiation – this process is a well-known cause of apoptosis activation in non-neoplasic tissues. 

 It has been shown that TNFα (tumoral necrosis factor alpha) induces apoptosis in vitro in tumoral lines and therefore a series of apoptotic events appearing in vivo could be attributed to the release of TNFα from infiltrating macrophages. In other instances, apoptosis can be the result of the attack of cytotoxic T lymphocytes on the malignant cells. Nevertheless, because there is an increase of apoptosis in preneoplasic locuses and in liver nodules that develop after the administration of chemical carcinogens it is unlikely that the above factors could be operative under these circumstances. It is possible that the regulatory mechanisms described above act in an early phase of the carcinogenesis process where increased apoptosis balances for the temporary rise in cell proliferation. 

 Finally, increased apoptosis in tumors can be an intrinsic characteristic of these cells because there are different apoptosis rates observed in similar tumor lines and this can be accounted for by the effects of different oncogenes. 
